# Hypothesis: Clues From Mammalian Hibernation for Treating Patients With Anorexia Nervosa

**DOI:** 10.3389/fpsyg.2018.02159

**Published:** 2018-11-12

**Authors:** Barbara Scolnick

**Affiliations:** Psychology and Brain Science, Boston University, Boston, MA, United States

**Keywords:** anorexia nervosa, hibernation, lipidomic profiling, ketogenic di, palmitoylethanolamide

## Abstract

This hypothesis is that anorexia nervosa (AN) is a biologically driven disorder, and mammalian hibernation may offer clues to its pathogenesis. Using this approach, this hypothesis offers suggestions for employing heart rate variability as an early diagnostic test for AN; employing the ketogenic diet for refeeding patients, attending to omega 3:6 ratio of polyunsaturated fatty acids (PUFAs) in the refeeding diet; and exploring clinical trials of the endocannabinoid-like agent, palmitoylethanolamde for patients with AN. This hypothesis also explores the role of lipids and autoimmune phenomena in AN, and suggest a lipodomics study to search for antibodies in the serum on patients with AN.

## Introduction

Anorexia nervosa (AN) is a severe disorder characterized by self-starvation, hyperactivity, and distorted body image (Kaye et al., 2009; American Psychiatric Association, 2013). The onset is often around puberty, and it is more common in females than males. The illness is difficult to treat, is frequently marked by relapses, and has the highest mortality of any psychiatric illness. It is associated with a high co-morbidity with substance abuse, especially alcoholism (Zipfel et al., 2000; Root et al., 2010). Recent data suggest patients with AN have an increased incidence of autoimmune disorders (Raevuori et al., 2014; Zerwas et al., 2017).

The pathogenesis and etiology are unknown, and thus developing effective treatments is very challenging. The framework for understanding the disorder has evolved from a primarily psychodynamic conflict (Bruch, 1978); to manifestation of Western Cultural pressure to be thin (Ahern et al., 2008); to a complex neurobehavioral disorder with strong evidence for underlying genetic vulnerability (Kaye et al., 2013; Yilmaz et al., 2015).

In 2003 Gusinger, advanced a very interesting hypothesis titled, “Adapted to Flee Famine: Adding an Evolutionary Perspective on Anorexia Nervosa” (Guisinger, 2003). She noted:

“The consensus of the field is that AN develops when psychopathology and social pressures to be thin act in concert with biological vulnerability. What if researchers have assumed the wrong direction of causality? Several lines of evidence, considered together, suggest that rather than psychological or medical pathology causing the bizarre behaviors and cognitions of AN, it is weight loss that leads to the symptoms. If the interpretation of the direction of causality is reconsidered, a number of discordant observations fall into place. The symptoms of AN are proposed to be biological responses to low body weight. AN is likely to result from a complex of adaptations selected in the prehistoric past when humans lived as sparsely distributed nomadic foragers. Although no longer adaptive, some individuals' bodies respond to very low body weight (caused by dieting or any reason) as though they must migrate from famine conditions” (Guisinger, 2003).

It is notoriously difficult to explore the circumstances at the onset of AN, because usually many months pass between the earliest changes in eating and the diagnosis of AN (Cottee-Lane et al., 2004). Although many qualitative studies have address the patients' experience of treatment for AN, there is a marked absence of reports of how the disorder took hold (Espindola and Blay, 2009; Bezance and Holiday, 2013; Sibeoni et al., 2017). Several books by recovered patients support the concept that benign weight loss preceded the psychological symptoms, rather than vice versa (Hornbacher, 1998; Arnold, 2013). Carrie Arnold, a science reporter who is also in recovery from AN noted:

“I spent the fall semester of my junior year studying in Scotland and returned carrying wonderful memories, gifts for all and yes, five extra pounds…I began the spring of my junior year with an innocent, earnest desire to work out regularly and shed those five pounds…Ten ponds came off before I even noticed…Looking back, I realized that I can say: there. That's when I first became anorexic. I wasn't clinically underweight at that point, but I was unable to start eating on my own, I was terrified of gaining weight, and I was unable to see what the issue was.” (Arnold, 2013).

With the hope that a radical reframing of the illness might open new avenues of treatment, this author makes the presumption that cultural and psychological issues make dieting more prevalent, but the etiology of AN is biologically driven. Another assumption is that weight loss from any cause including benign dieting, activates a series of metabolic signals that cascade into self-starvation, and hyper-exercise in genetically susceptible individuals.

Metaphors draw attention to similarities between two separate, seemingly unrelated entities, and while innately oversimplified, can be powerful tools to illuminate and clarify issues that otherwise are lost in complexity. In that spirit, and borrowing from Gusinger's hypothesis, this author offers a metaphor between AN and mammalian hibernation (Scolnick and Mostofsky, 2014). The latter is an extreme metabolic adaptation to food depletion and cold, and AN is a metabolic mal-adaptation to transient decreased food intake. Much about hibernation remains a mystery, but it is becoming clear that the signals to enter hibernation in the autumn, and emerge in the spring are not dependent on unique metabolic pathways. Rather, hibernation signals use the same metabolic pathways present in all mammals, including humans (Schwartz and Andrews, 2013).

While not claiming this is the “whole picture” this metaphor is consistent with some features of AN—the geographic pattern showing high prevalence in temperate climates, and rarity in the tropics (Njenga and Kangether, 2004; Hoek et al., 2005; Vazquez et al., 2006; Gutierrez et al., 2017), the subtle worsening in winter months (Favaro and Santonastaso, 2009; Fraga et al., 2015), the frequently observed hypothermia (Nishita et al., 1985), and the tendency for many patients to cross over from pure restriction to binging which resembles the hibernating mammal's seasonal pattern of binging in late summer and autumn followed by starvation in winter (Eddy et al., 2008).

## Methodology

This is presented solely as a hypothesis andz is not an exhaustive literature review. In a sense the methodology was to approach the disorder of AN with no preconceptions except the assumption that It is biologically driven, then to read widely, and to see if any coherent themes emerged from disparate areas. The hibernation theory emerged after reading the literature on changes in fatty acids in AN, and noting similar studies in hibernation. Public Med was the only source used, and the method for finding studies was totally theoretical. Oftentimes one article would lead to many more. Some topics, such as heart rate variability in AN, ketogenic diet in AN, or endocannabinoid changes in patients with AN had less than 20 articles, thus all papers referenced in Public Med were reviewed, and all the relevant articles are referenced in the text. Other topics, such as the physiology of endocannabinoids (McPortland et al., 2014; Maccarrone et al., 2015), eicosanoids (Dennis and Norris, 2015), soluble epoxide hydrolase (sEH) (Swardfager et al., 2018), and lipodomic analysis (Sethis and Brietzke, 2017) have a vast literature. For the interested reader, there are current recent reviews of these fields, and this author is not an expert on these areas.

### Bradycardia in hibernation and heart rate variability in AN

It has long been known that black bears are immobile during hibernation, but if disturbed can wake up in seconds, and mount a vigorous defense. Elegant research implanting bears with pacemakers before releasing them back in the wild, have documented that extreme bradycardia is the norm while hibernating (Laske et al., 2011). Heart rates average 8 beats/min in winter and 130 beats/min in the summer. The longest period of asystole was 14 s with a breathing rate of 1.53 breaths/min. Respiratory sinus arrythmia dampening proved to be a very sensitive measure of arousal, noted before any behavioral change or increase in pulse. Respiratory sinus arrhythmia refers to the oscillations between breathing and heart rate, which are present in all mammals (Grossman and Taylor, 2007). Normally heart beats increase during inspiration and decrease during expiration. Exaggeration of this pattern reflects increased vagal tone, and correlates with bradycardia. Statistical analysis of the oscillations formed by plotting breathing rate and heart rate over time, derived from a 15 min EKG, is a field of physiology known as heart rate variability (Billman et al., 2015). Heart rate variability is a more sensitive indicator of vagal tone than simply measuring the pulse.

Clinically, it is widely known that patients with AN demonstrate bradycardia, with estimates that 70% of patients show heart rates <50 beats/min at presentation (Yaham et al., 2013). At least 15 studies have examined heart rate variability parameters in patients with AN, and almost all found high values for respiratory sinus arrhythmia (Galetta et al., 2003; Cong et al., 2004; Platisa et al., 2006; Muraldo et al., 2007; Dhoble et al., 2008; Ishizawa et al., 2009; Mazurak et al., 2011a; Palova et al., 2012; Jacoangeli et al., 2013; Bomba et al., 2014). The few studies that did not find elevated respiratory sinus arrythmia, included patients in various states of recovery (Vigo et al., 2008). A recent literature review noted the lack of consistency and standardized practices in measuring heart rate intervals, yet the general finding that AN is a state of increased RSA is strong (Mazurak et al., 2011b). These findings are a bit of a paradox because, in many studies, higher heart rate variability and respiratory sinus arrythmia has been associated with improved cardiac health (Buccelleti et al., 2009), general good health (Tsuhi et al., 1994), and emotional resilience (Hildebrandt et al., 2016). It is interesting that oftentimes patients with AN superficially appear to be healthy, especially at the onset of the disorder before the cachexia becomes evident. Heart rate variability might be a useful tool in diagnosing early onset AN.

### Ketogenic diet and AN

**Figure 1:**
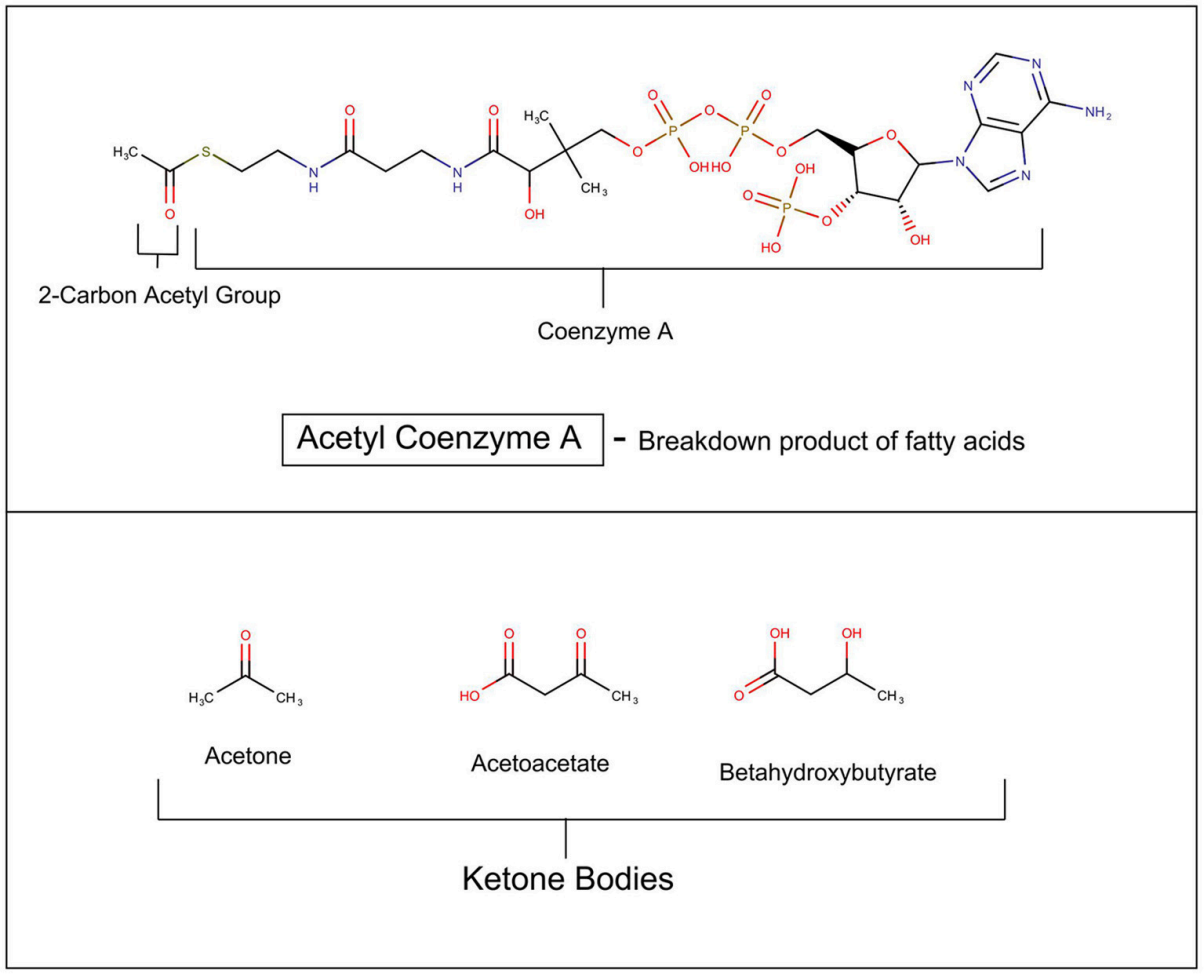
Ketosis.

In mammals, glucose is the major source for cellular fuel under most conditions, but in states where cellular glucose is insufficient such as untreated diabetes mellitus, fasting, semi-starvation, extreme exercising, and notably hibernation, the metabolism switches to using more fat (Andrews et al., 2009). In these situations, fatty acids are broken down in the mitochondria into acetyl Co-A, which enters the Krebs cycle to produce cellular energy. The liver begins to synthesize beta hydroxyacetate, acetoacetate, and acetone from the excess acetyl-CoA (see Figure 1). These three compounds are collectively called ketone bodies. It has recently been discovered that astrocytes also have the capacity to generate ketones (Le Foll and Levin, 2016). An elegant and daring study by Owens and Cahill in 1967 of in-hospital monitoring of three obese patients during a 5–6 week fast, including multiple catheterization of liver and brain, demonstrated that after 3 days of starvation the metabolism of the brain undergoes radical transformation (Owen et al., 1967). Beta hydroxybutyrate and acetoacetate replace glucose as the primary fuel.

Patients with AN, subsist on a semi-starvation diet, and often have elevated serum ketones. This raises the issue of whether the state of ketosis might be metabolically, and psychologically rewarding for patients (Scolnick, 2017). This would be important because it is possible to reproduce the ketotic state without starvation, by invoking the ketogenic diet.

The ketogenic diet is a very high fat, low carbohydrate diet that continues to provide adequately nourishment while the metabolism becomes ketosis driven. It replicates starvation, but the individual does not starve. It has great scientific interest because it is an effective treatment for pediatric seizures (Wilder, 1921; Freeman et al., 2009), and has proven to be safe and surprisingly well-tolerated, when both the child and parent see the results (Freeman et al., 2007). Recently small pilot studies suggest it might be effective for neurodegenerative disorders such as Parkinsons and Alzheimers diseases (Vanitallie et al., 2005; Henderson, 2008).

Two animal clinical trials, one from 1971 and one from 2008 demonstrated that a very high fat diet interrupts self-starvation in a rodent model of anorexia (Barbotiak and Wilson, 1971; Brown et al., 2008). The activity based anorexia model, although not perfect, is considered the best animal model because it replicates the self-starvation, hyperactivity, and changes in leptin, ghrelin, and cortisol seen in human AN (Gutierrez, 2013). The model is induced by housing the rodent in a cage with *ad lib* access to a running wheel, and exposing it to slightly restricted feeding times. The rodent who develops activity based anorexia starts to run excessively, lose weight, and self-starve.

There are no human trials of the ketogenic diet in patients with AN.

### Omega 3: omega 6 ratios in hibernators and in AN

Current research shows that lipid pathways especially polyunsaturated fatty acids (PUFAs) are major signaling agents in hibernation (Geiser and Kenagy, 1987; Geiser, 1990; Hill and Florant, 2000; Gerson et al., 2008; Ruf and Arnold, 2008; Arnold et al., 2011). For decades, isolated clinical and research studies have reported abnormalities in lipid metabolism in patients with AN (Halmi and Fry, 1974; Holman et al., 1995; Curatola et al., 2004; Zák et al., 2005; Swenne et al., 2011). Within the framework of the metaphor, this author presents a highly speculative theory that lipid pathways are key to causing and perhaps treating AN.

**Figure 2:**
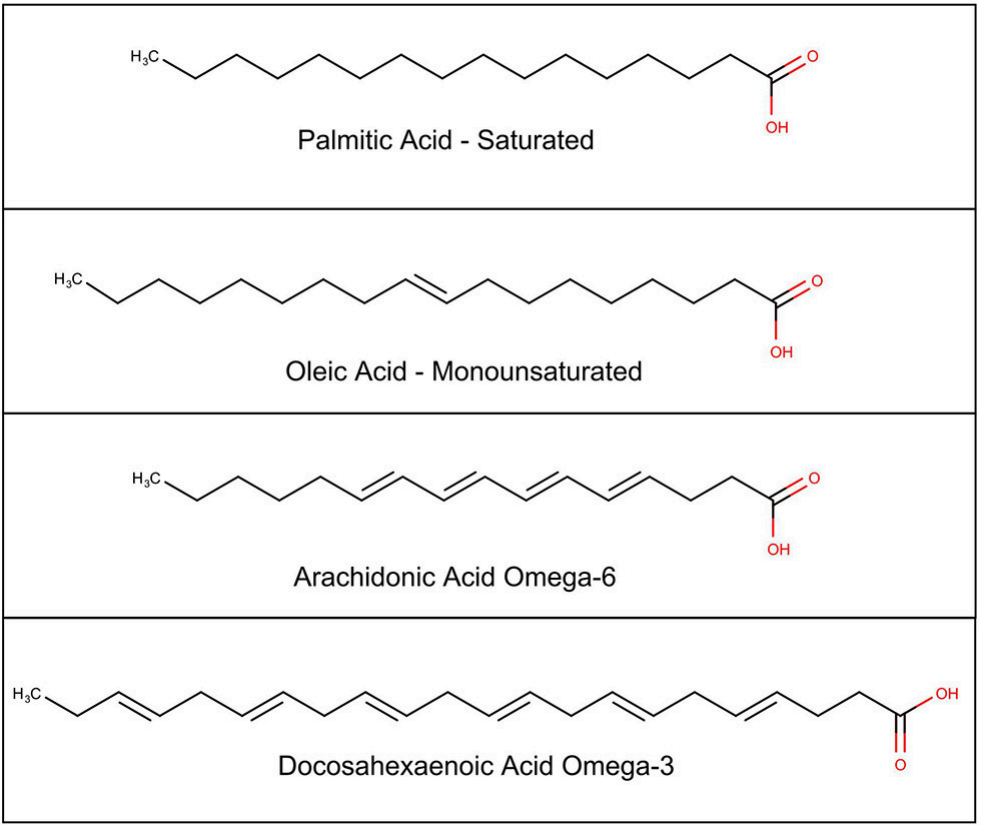
Chemical structure of selected fatty acids.

A brief review of the complex biochemistry of lipids, shows that the building blocks for most complex lipids are the fatty acids, which are classified as short-chain if there are 2–6 carbons; medium-chain if there are 6–12 carbons; or long-chain if there are 14 or more carbons. They are further classified by the number of double bonds, which gives the chain more flexibility. Saturated fatty acids have no double bonds; monounsaturated fatty acids have one double bond; and PUFAs have more than one double bond (see Figure 2).

The PUFAs can be divided into several families, based on where along the carbon chain, the double bonds occur (see Table 1). These are referred to as omega families, “omega” signifying “backwards” because the carbon at the methyl end of the chain is numbered carbon 1, which is backward from classical chemical numbering which numbers the carbons from the acidic end (Holman, 1964). The omega 3 fatty acids first double bond is at the third carbon; the omega 6 fatty acids double bonds start at carbon 6. Two of these fatty acids are essential in humans, alpha linolenic acid from the omega 3 family, and linoleic acid from the omega 6 family. These essential fatty acids must be obtained from the diet. A series of elongation and desaturation enzymes act on these fatty acids to derive the remainder of the fatty acids in both families. Longer chain PUFAs can also be obtained from the diet, but because they can also be metabolically synthesized, they are not considered essential.

**Table 1:** Omega fatty acids.

**Name**	**Abbreviation**	**Omega name**
**OMEGA-6 FATTY ACIDS FAMILY**		
Linoleic acid	LA	18:2n-6 (essential)
Gamma linolenic acid	GLA	18:3n-6
Dihomo-gama-linolenic acid	DGLA	20:3n-6
Arachidonic acid	AA	20:4n-6
Adrenic acid		22:4n-6
Tetracosatetraenoic acid		24:4n-6
Tetracosapentaienoic acid		24:5n-6
Docosapentaenoic acid	DPA (n6)	22:5n-6
**OMEGA-3 FATTY ACIDS FAMILY**		
Alpha linolenic acid	ALA	18:3n-3 (essential)
Stearadonic acid	SDA	18:4n-3
Eicosatetraienoic acid	ETA	20:4n-3
Eicosapentaenoic acid	EPA	20:5n-3
Docosapentaenoic acid	DPA n3	22:5n-3
Tetracosahexaenoic acid		24:6n-3
Docoshexenoic acid	DHA	22:6n-3

*Omega labeling is Number of Carbons:Number of Double Bonds:n-6 omega family or n-3 omega family*.

Mammals cannot convert omega 3 fatty acids to omega 6, and vice versa. Thus, a constant theme in PUFA metabolism is the competition between the omega 3 and omega 6 families, as they share the same metabolic pathways.

The ratio remains an intriguing window into metabolic changes, and once again it is worthwhile to examine the hibernating animal. Several studies have noted dramatic seasonal oscillations in serum fatty acids, with respect to degree of saturation, and the ratio of omega 3:6 fatty acids in hibernators (Ruf and Arnold, 2008; Arnold et al., 2011). In late autumn, when the weather cools, the phospholipid makeup of cellular membranes in the heart become enriched with omega 3 highly unsaturated fatty acids, and when the animal emerges from hibernation the ratio reverses. An evolutionary highly conserved adaptation to ambient cold, seen from bacteria to mammals is that the cell membrane maintains its viscosity by altering proportions of fatty acids (Ernst et al., 2016).

Two studies, one of normal adults living in Antwerp Belgium, and another of patients with seasonal affective disorder in California have shown humans also experience seasonal variation in PUFAs and omega 3:6 ratios (DeVriese et al., 2004; Hennebelle et al., 2017). In both these studies the oscillations were much more subtle than those seen in hibernators, but nevertheless present.

As far as AN, multiple studies have consistently demonstrated very profound distortions in serum PUFA profiles, compared to normal, although there are no studies focusing on seasonal changes. Interestingly, most of these have found a skewing toward more omega 3 fatty acids over omega 6, which is opposite from changes seen in populations eating the Western diet. This might reflect the preference for salads, and avoidance of meat, observed in many patients with AN.

In the last few decades there has been a lot of discussion about how the Western diet has become skewed from our traditional diet, and this might be contributing to heart disease, diabetes, obesity, and other disorders (Simopoulous, 2016). Omega 3 fatty acids are plentiful in green leafy vegetables, and wild fish; whereas seeds and meats are rich in omega 6 fatty acids. Many studies have shown that the Western diet has shifted from a balanced omega 3:6 ratio to one that heavily favors omega 6 fatty acids over the omega 3 family (Blasbalg et al., 2017). At one point, there was enthusiasm that supplementing the Western diet with pills containing omega 3 fish oils would lead to improved cardiac health. As larger public health studies have been conducted, there are more negative results, thus unhappily confirming that lipid metabolism is complex, and simply resupplying a fatty acid, akin to vitamin replacement for classical nutritional deficiency disorders is not effective (Rice et al., 2016). Similarly, a pilot trial of omega-3 supplementation with patients with AN was disappointing although there was a possible mild decrease in anxiety (Ayton et al., 2004).

Shih et al. has focused on the omega 3:omega 6 refeeding ratio in patients with AN with a nuanced approach targeting a specific enzyme named sEH (Shih et al., 2017).

**Figure 3:**
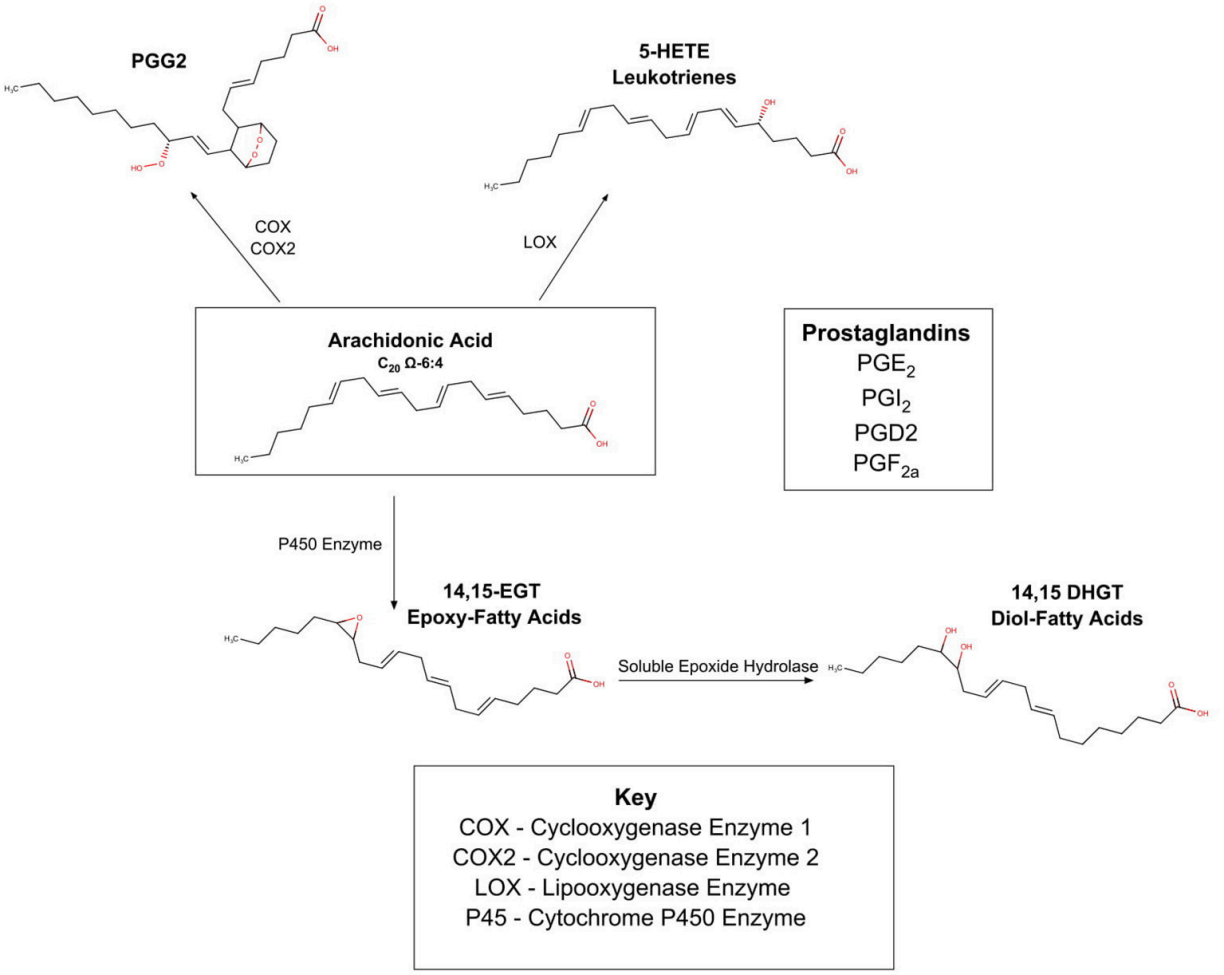
Oxidation of PUFA's by COX, LOX, P450.

To understand the implications of this pathway, Figure 3 summarizes the complex web of PUFA enzyme oxidation products. There are three known oxidative enzymes:

cyclooxygenase(COX);
lipooxenase (LOX);
cytochrome P-450 enzymes (CYP).

As shown in Figure 3, these enzymes act upon the PUFAs (in Figure 3 arachidonic acid is depicted), and form a myriad of compounds commonly known as the prostaglandins and leukotrienes. These products are present in most cells of the body and are locally active and short acting. It was originally thought that the 20-carbon omega 6 PUFA, arachidonic acid was the precursor to all the compounds in this family, and this pathway is therefore often referred to as the eicosanoid (eicos is Greek for 20) pathway. Recent work suggests many PUFAs from both the omega 3 and omega 6 families are subject to enzymatic oxidation (Spector and Kim, 2015). The innate competition between the omega 6 and omega 3 families is important in this area, as it is becoming clear that different PUFAs are oxidized to different prostaglandins. The different prostaglandins have different properties which are frequently antagonistic; some are inflammatory while others are anti-inflammatory; some are vasoconstrictor and others are vasodilators.

In 2014, a preliminary genetic study discovered that a gene coding for sEH protein was associated with an increased risk of AN (Zeeland et al., 2014). Soluble epoxide hydrolase (sEH) acts on epoxy eicosatienoic acids, transforming them into diols. As shown in Figure 3, epoxy eicosatienoic acids, in turn are produced when PUFA are oxidized via the Cytochrome P450 pathway. Shih performed a lipodomic and metabolomic comparison of the serum from 36 healthy controls, 30 ill patients with AN, and 30 “recovered” patients with AN, measuring PUFAS as well as 80 oxidation products (Shih et al., 2016). Results showed that ill patients had high sEH product/substrate ratios implying increased activity; and “recovered” patients also showed increased sEH activity compared to controls. She also noted that omega 6:omega 3 PUFA profile was lower in ill patients than in controls, and that the ratio was inversely associated with anxiety in patients with AN. She is thus exploring whether the epoxide and subsequent diol derived from omega 6 PUFAs have different physiology from the epoxide and subsequent diol derived from the omega 3 PUFAs, and whether this can cause the patient to avoid certain foods that promote a subtle inflammatory state (Shih, 2017).

### The endocannabinoid system (ECS)

**Figure 4:**
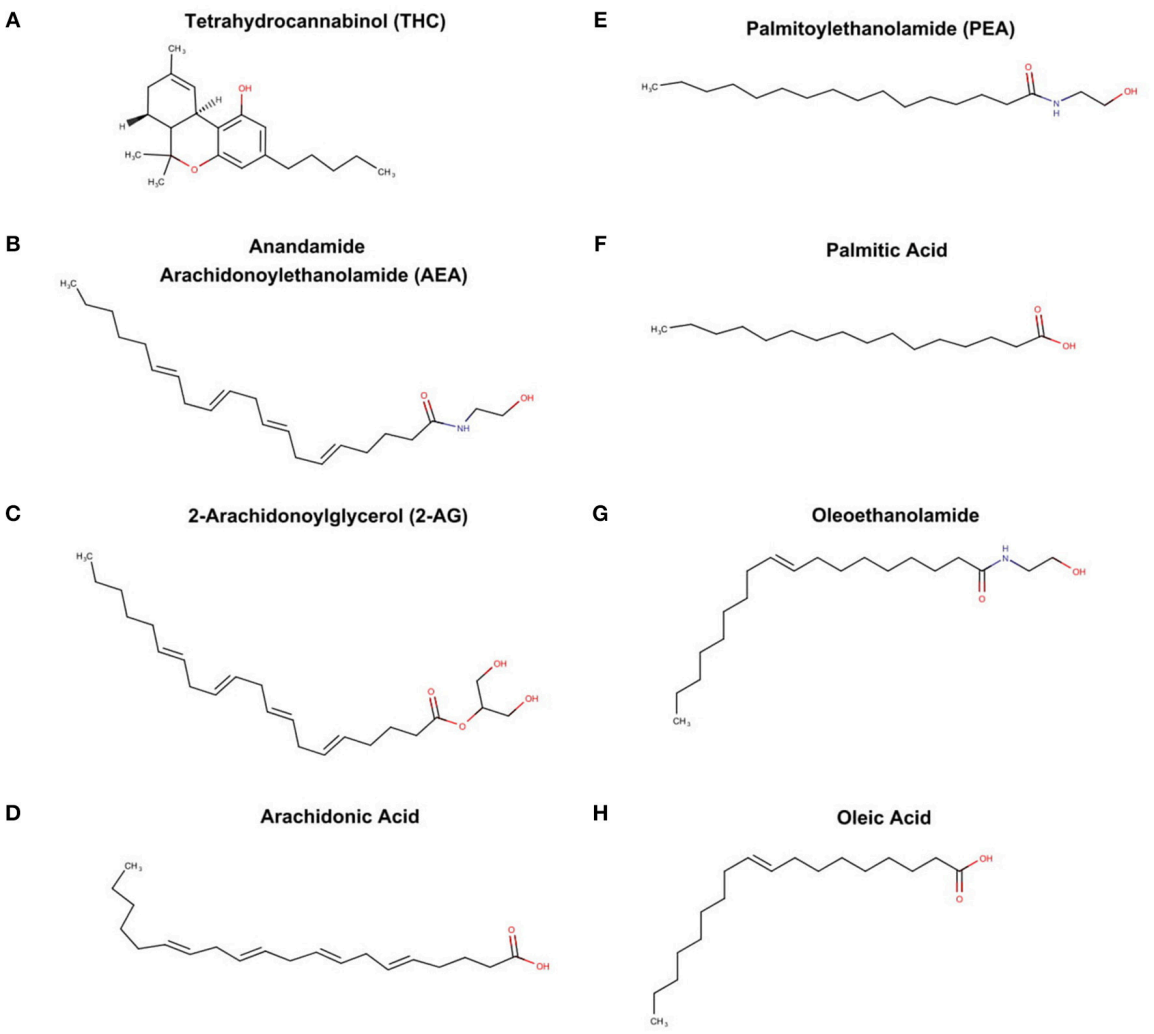
THC from marijuana plant, endocannabinoid, and endocannabinoid-like agents **(A)** tetrahydrocannabinoid (THC), **(B)** anandamide arachidonoylethanolamide (AEA), **(C)** 2-arachidonoylglycerol (2-AG), **(D)** archidonic acid, **(E)** palmitoylethanolamide, **(F)** palmitic acid, **(G)** oleoethanolamide, and oleic acid **(H)**.

The endocannabinoid system is also a byproduct of PUFA metabolism, although the discovery was independent of the prostaglandin pathways. Raphael Mechoulan's laboratory isolated and chemically characterized tetrahydrocannabinoid (THC) as the active ingredient in marijuana in 1964 (Mechoulam et al., 1967) (see Figure 4A), but it was not until 1998 that THC was demonstrate to stimulate specific receptors in the brain and periphery of humans (Devane et al., 1988). These receptors were characterized and labeled cannabinoid receptor 1 (CB1) and cannabinoid receptor 2 (CB2). Subsequently, it was found that endogenous compounds derived from arachidonic acid were able to also stimulate these identical receptors (Devane et al., 1992; Mechoulam et al., 1995). These “endocannabinoids” known chemically as arachidonoylethanolamde (AEA) or more commonly referred to as anandamine (Sanskrit for bliss) (see Figure 4B); and 2-arachidonooylglycerol (2AG) (see Figure 4C) are very different chemically from tetrahydrocannabinol (THC), the active agent in marijuana. While only anandamide and 2AG are known to stimulate the CB1 and CB2, other fatty acid derived ethanolamides (see Figures 4E,G) act in concert with anandamine and 2AG, and thus are referred to as “endocannabinoid-like agents” (Ho et al., 2008). It is important to note that the classic endocannabinoids, and the “endocannbinoid-like agents” are derived from the polyunsaturated fatty acids, arachidonic acid (see Figure 4D) and palmitic acid (see Figure 4F) respectively.

Analogous to prostaglandins, these compounds act locally, are short acting, and set in motion myriad and sometimes antagonistic effects. While much remains unknown, it is clear the ECS is intimately involved with very basic physiology such as circadian rhythms, feeding behaviors, pain sensation, and reward feelings (Vaughn et al., 2010; Lu and Mackie, 2018). Adding to the complexity, anandamine, and 2AG are subject to oxidation by the same enzymes that are part of the eicosanoid pathways, and thus there is potential for tremendous cross-talk between prostaglandins and endocannabinoids (Rouzer and Marnett, 2011).

Monteleone et al. has examined the ECS in patients with AN (Monteleone et al., 2005, 2012, 2015). The pattern of serum endocannabinods differs between normal controls, patients in recovery from anorexia, and patients with acute anorexia, especially when subjects were presented with a task of eating a favorite food when sated, which is described as hedonic eating.

A recent encouraging study, employing the activity based anorexia model found that pretreatment of rats with THC or a synthetic CB1 agonist significantly attenuated the excess running, and starvation in the affected rodents and, normalized leptin and cortisol profiles (Scherma et al., 2017).

Because the ECS is complex, and not completely mapped there is a great deal of risk using pharmaceuticals to manipulate the system. This is illustrated by the case of Rimonobant, a CB1 antagonist that had proven effective in preliminary trials in inducing weight loss in obese patients (Sam et al., 2011). Although it was never approved in the United States, it was marketed in Europe in 2006, and removed worldwide in 2008 when a significant increased incidence of depression and suicide was noted in patients prescribed Rimonobant.

Palmitoylethanolamide, commonly called PEA is a fatty-acid-ethanaolamide derived from the 16 carbon saturated palmitic acid that is plentiful in palm oil. PEA was isolated from egg yolks in 1957, and reports of its use medically for various inflammatory conditions date from the late 1960s (Hesselink, 2013). It is produced in Europe as a regulated nutraceutical, and easily available on the internet. While some have advocated for pilot studies in patients with AN, there have been no clinical trials (Scolnick, 2018).

### Phospholipids oxidation products as epitopes for auto antibodies

There has long been a clinical impression that many patients with AN develop autoimmune diseases. Two recent epidemiological studies from Scandinavia confirm the association of autoimmune disorders and AN. A 2014 study from Helsinki followed all 2,342 adolescents hospitalized for eating disorders from 1995–2011 and found 8.9% developed an autoimmune disorder compared to an expected 5.4% (Zerwas et al., 2017). A study from Denmark following 1 million children and adolescents found that adolescents with AN had a 64% higher hazard ratio for autoimmune/inflammatory disease than normal controls (Raevuori et al., 2014).

Clinically, a possible association has been noted between pediatric autoimmune disorder associated with streptococcus (PANDAS) and isolated cases of AN (Calkin and Carandan, 2007). The proposed pathophysiology is that antibodies develop to the streptococcus and cross-react with brain tissue, similar to the situation of acute chorea associated with rheumatic fever. Likewise, clinicians have commented on the co-occurrence of AN and systemic lupus erythematosis (Toulany et al., 2014).

A genome-wide association study (GWAS) comprising 3,495 AN cases and 10,982 controls, found one highly significant locus on chromosome 12 (rs4622308) which has been noted on GWASs for rheumatoid arthritis and type 1 diabetes; and regions surrounding the locus region has been implicated in other autoimmune disorders (Duncan et al., 2017).

The evidence for autoimmune antibodies in AN is very preliminary. Fetissov et al. found that patients with AN and bulimia nervosa have autoantibodies against two hypothalamic peptides involved in hunger/satiety; alpha-melanocyte-stimulating-hormone and ACTH, in a pattern that differs from normal controls (Fetissov et al., 2002). They also noted that anxiety and obsessions, core traits of ill patients with eating disorders, correlate with the level of autoantibodies in the serum (Fetissov et al., 2005).

It is intriguing to imagine that 1 day AN might be managed as an autoimmune disorder. While highly speculative, we note the emerging science linking oxidation products of PUFAs to pathological autoantibodies. Briefly, in addition to enzyme-linked PUFA oxidation, via the COX, LOX, and CYP enzyme systems, lipids are also subject to oxidation by non-enzymatic reactions. These reactions tend to be self-generating and lead to formation of lipid hydroperoxides, and reactive aldehydes, which can react with neighboring proteins (Kurien et al., 2006). It has been suggested that these oxidation-derived-protein complexes, can function as epitopes (antibody-inducers) and lead to antibodies against self-proteins. Using a murine model of systemic lupus erythemtosis, Otaki et al. found antibodies that cross reacted both to DNA, which is the hallmark diagnostic test for lupus, and an aldehyde product arising from peroxidation of an omega 6 PUFA (Otaki et al., 2010). Quintana et al. recently reported on using lipodomic analysis and antigen microarrays to discover autoantibodies to lipid products that can be used as biomarkers for multiple sclerosis (Quintana et al., 2012).

Linking PUFA abnormalities in patients with AN to autoimmune processes has not been investigated.

### Implications of the hypotheses

This speculative hypothesis, focusing on an analogy between AN and rogue hibernation has given rise to four specific suggestions for exploratory studies.

First, a pilot study comparing the HRV of patients with new onset AN with normal healthy fit adolescents might lead to a non-invasive test for early diagnosis and screening of at-risk populations such as student athletes.

Second, a pilot trial of ketogenic diet in refeeding from AN is suggested. The animal data obtained from activity based anorexia model is encouraging, and the diet is in clinical use for pediatric seizures, and is safe for this population.

Third, a pilot trial of palmitoylethaloamide, an endocannabinoid-like agent, in patients with AN is also suggested. Data from the activity based animal model is very encouraging, although THC and a synthetic CB1 agonist were used, not palmitoylethanolamide. As noted, synthetic agents commonly have untoward effects, and would require extensive study, but palmitoylethanolamide has a long track record of safety.

Fourth, a pilot study of serum from patients with acute AN, recovered AN, and normal controls using lipidomics and antigen arrays, akin to the referenced study of patients with multiple sclerosis, might lead to identification of biomarkers.

Returning to the analogy with hibernation, there are currently efforts underway to apply the techniques of lipidomics, genomics, and proteinomics to decipher the metabolic signals of hibernation (Vermillion et al., 2015). It might be a very opportune time to share information across divergent fields of study in an attempt to find treatments for AN.

## Author contributions

The author confirms being the sole contributor of this work and has approved it for publication.

### Conflict of interest statement

The author declares that the research was conducted in the absence of any commercial or financial relationships that could be construed as a potential conflict of interest.
